# Sun protection during active travel on foot or by (e-)bike: an exploratory nationwide cross-sectional study from Germany

**DOI:** 10.3389/fspor.2026.1957202

**Published:** 2026-10-14

**Authors:** Lisa Voß, Tatiana Görig, Anne Kerstin Kubbe, Clara Tristram, Katharina Diehl

**Affiliations:** 1 Department of Medical Informatics, Biometry and Epidemiology, Friedrich-Alexander-Universität Erlangen-Nürnberg (FAU), Erlangen, Germany; 2 Department of Sport Science and Sport, Friedrich-Alexander-Universität Erlangen-Nürnberg (FAU), Erlangen, Germany; 3 Bavarian Cancer Research Center (BZKF), Erlangen, Germany; 4 Comprehensive Cancer Center Erlangen-European Metropolitan Area of Nürnberg (CCC ER-EMN), Erlangen, Germany

**Keywords:** active travel, children, commuting, Germany, skin cancer, sun protection, sunscreen

## Abstract

**Background:**

Solar ultraviolet (UV) radiation is the primary environmental risk factor for skin cancer. Consistent sun protection is therefore recommended for prevention. We examined the extent to which people protect themselves (and accompanied children) from the sun during active travel for transport reasons.

**Methods:**

We used data from the wave 2025 of the National Cancer Aid Monitoring online (NCAM-online), a nationwide online survey in Germany. For our analysis with focus on walking/cycling for transport, 3,264 individuals were included. We stratified our analysis by reason for traveling and analyzed sun protection in the subgroup of participants who actively traveled with children.

**Results:**

The most common sun-protection measures were wearing a shirt with sleeves covering the shoulders (52.1%), wearing sunglasses (46.8%), and traveling in the shade (38.5%). Sunscreen and headwear were used less frequently. When children were accompanied, they were more likely to be protected than the accompanying adult. Under perceived increased UV conditions, 42.8% opted not to walk or cycle.

**Discussion:**

Although our findings indicate that children with their more UV-vulnerable skin are more likely to be protected and that some participants forgo active travel under increased UV conditions, sun-protection practices during active travel leave room for improvement. Both individual-level education and structural measures in urban planning (e.g., increasing shade provision) appear warranted.

## 1 Introduction

While sunlight is essential for human beings, its ultraviolet (UV) radiation is a major risk factor for the development of skin cancer (1, 2). UV radiation can lead to both keratinocyte carcinoma and malignant melanoma (1, 3). The incidence of both cancers has steadily increased in recent years in Western industrialized nations (3–5).

Consistent use of sun protection measures is essential for the primary prevention of skin cancers (6–8). These measures include avoiding intense UV exposure by seeking shade, wearing clothing that covers skin exposed to the sun, and applying sunscreen to uncovered skin (9–11). These evidence-based measures are particularly important for children and adolescents (9, 10). However, sun protection behavior in the general population is sometimes inadequate (12–14). This applies to both adults (14, 15) and children (16–20). Low levels of sun protection are also observed in specific subgroups, such as outdoor workers (21, 22), or in particular situations, like outdoor sports (23, 24). One everyday situation that has received little research attention yet is sun protection during active travel on foot or by (e-)bike.

Active travel or active mobility on foot or by (e-)bike (or any other human powered vehicle or active mode of transport) describes situations of active transport where people travel from one place to another to run errands or to get to work, school, or leisure activities (25, 26). This may happen on a regular or situational basis. Active travel also includes accompanying children to school or recreational facilities (27). In these situations, sun protection is important, especially during the summer, as children's skin is sensitive towards the UV radiation (28).

Since there has been little research on this topic so far, the aim of this study was to explore the use of recommended sun protection measures while active traveling on foot or by (e-)bike during the summer. We addressed the following research questions:

- How often do people use different sun protection measures during active travel on foot or by (e-)bike for everyday distances in summer?
- Does sun protection behavior differ by trip purpose (e.g., active travel with or without children) and by sex/gender?
- To what extent are children protected from the sun during active travel? Do children's sun-protection practices differ from those of the accompanying adults?

Answers to these questions from this exploratory study can help us better understand sun-protection behavior during active travel and develop targeted recommendations for education and skin cancer prevention.

## 2 Methods

### 2.1 Study setting

The data for this exploratory study were drawn from the tenth wave of the National Cancer Aid Monitoring (NCAM-online) (29). The study population included 4,304 participants aged 16–65 years from Germany, who were recruited via an online panel of the ISO-certified international survey company Bilendi (https://bilendi.de). The recruitment was quota-based according to sex/gender, age, educational level, and federal state. The online-survey took place in November 2025. All individuals gave informed consent for participation. Ethical approval for this study was granted by the Ethics Committee of the Friedrich-Alexander-Universität Erlangen-Nürnberg (22-337-S on November 2nd 2022, and 22-337-2-S on October 14th 2025).

### 2.2 Measures

To gather information about the participants’ active travel behavior, we used a multi-staged process. First, they were asked ‘In summer, how often do you make everyday trips of 10 min or longer on foot or by (e-)bike?’. This question could be answered with ‘never’, ‘from time to time’ or ‘often’. Second, those who indicated to take such distances from time to time or often were asked ‘For what reasons do you take these distances?’ to validate whether they belong to our target population. Participants could select more than one option of the following: ‘Active travel to or from work/educational facility’, ‘Active travel to or from leisure activities (e.g., going to sport facilities, visiting friends)’, ‘To run errands (e.g., going shopping, going to doctor's appointment)’, ‘To accompany children, to drop them off or pick them up’, ‘As leisure activity’, and ‘Walking the dog’. Those who selected only one of the last two response categories were excluded from further analyses, as they did not engage in active travel for transport reasons according to our definition. Our focus was on walking/cycling for transport and not on walking/cycling for recreation (30).

Participants, who responded to be engaged in active travel to and from work/educational facility, to and from leisure activities, and to run errands were then asked about use of sun protection measures during their travel. The following measures were addressed:

- ‘Application of sunscreen or day cream with sun protection factor on the face’,
- ‘Application of sunscreen on the remaining areas of the body exposed to the sun’,
- ‘Wearing a top with sleeves covering the shoulders’,
- ‘Wearing headwear (e.g., hat, cap, helmet)’,
- ‘Wearing sunglasses’, and
- ‘Traveling the distances in the shade’.

Participants could respond using categories ‘never’, ‘rarely’, ‘sometimes’, ‘often’ or ‘always’, as it has been recommended by Glanz et al. (31). There was no “not applicable” option available. Participants who reported traveling with children were asked about their own and the children's use of sun protection measures listed above during the travel. For further analyses, we dichotomized the answer categories into never/rarely/sometimes vs. often/always to reduce complexity.

To address the behavioral adaptation in travel in response to increased solar radiation in summer, all participants engaged in active travel were asked ‘Does it ever happen that, because of high solar radiation in summer, you choose not to travel on foot or by bike/e-bike and instead take a car or bus/train?’. Further, participants answered the question ‘Does it ever happen that, because of high solar radiation in summer, you choose to use additional sun protection measures when traveling by foot or by bike/e-bike?’. Both questions could be answered with ‘yes’ or ‘no’.

Prior the use in the survey, all items on active travel and use of sun protection during active travel were examined in a cognitive pre-test involving 15 individuals, in order to improve their comprehensibility and optimize the answer categories.

The participants’ sociodemographic information included gender identity (male/female/divers), age, immigrant background [yes/no, based on questions following Schenk et al. (32)] and partnership status (yes/no). Due to small numbers, participants who identified as “diverse” were excluded from sex-stratified (female/male) analyses but are included in the overall frequency counts. In addition, information on the participants’ highest level of school education (low/medium/high) and occupational status (fulltime/parttime/none) were gathered. Furthermore, the self-reported skin type was surveyed using the classification by Fitzpatrick (33).

### 2.3 Statistical analysis

We conducted descriptive univariate and bivariate analyses using Chi squared Test. For paired comparisons we used McNemar Test. Given the exploratory nature of this study and the absence of pre-specified hypotheses, we did not adjust for multiple testing. All analyses were conducted with IBM SPSS Statistics (Version 29; IBM Corp.). A predefined significance level of 0.05 was used; *p*-values below 0.05 were considered statistically significant.

## 3 Results

After excluding participants who did not engage in active travel during summer (*n* = 491) and those who walked/cycled solely for recreation (*n* = 549), the final sample included 3,264 individuals reporting active travel for different purposes (Supplementary Figure S1). Of these, 52.0% were male, 47.7% female, and 0.3% diverse (Supplementary Table S1). The mean age was 42.4 years (SD: 13.4). An immigrant background was reported by 9.1% of the participants. The educational level was rated low for 24.3%, medium for 30.7%, and high for 45.0% of travelers. Regarding occupation, 58.3% of all travelers worked fulltime, 19.2% worked parttime and 22.2% were unoccupied. Approximately two thirds of all travelers had a partner (65.9%). Skin types I and II were self-reported by 40.6%, and skin types III to IV by 59.4%.

Supplementary Table S1 also gives an overview of the subgroups of active travelers. Because participants could report multiple reasons for active travel, the groups are not mutually exclusive and may overlap. The mean age was 40.3 years (SD 10.4) among participants who traveled with children and 42.5 years (SD 13.7) among those who traveled for other reasons.

### 3.1 Sun protection during active travel to work/educational facility, leisure activities, and to run errands

In the group of those who traveled to work/educational facility, leisure activities, or to run errands, wearing a shirt with sleeves that covers the shoulders was the most frequently used sun protection measure (52.1%) followed by wearing sunglasses (46.8%), traveling distances in the shade (38.5%), application of sunscreen on the face (32.5%), wearing headwear (29.5%) and application of sunscreen on the remaining areas of the body exposed to the sun (26.6%, Table 1). For all measures, we found significant differences between men and women. While women were more likely to wear sunglasses (49.5% vs. 44.4%, *p* = 0.016), travel distances in the shade (43.8% vs. 33.8%, *p* < 0.001), apply sunscreen on face (43.7% vs. 22.2%, *p* < 0.001) and body (31.6% vs. 22.0%, *p* < 0.001), men were more likely to report wearing a shirt with sleeves covering the shoulders (54.6% vs. 49.4%, *p* = 0.013), and headwear (37.5% vs. 20.8%, *p* < 0.001).

**Table 1:** Sun protection use during active travel to work/educational facility, leisure activities, and to run errands.

Sun protection behaviors	All travelers, who travel to or from work, leisure activities, or to run errands (*n* = 3,186)				Travel to work/educational facility (*n* = 1,494)				Travel to leisure activities (*n* = 1,673)				Travel to run errands (*n* = 2,454)			
	Total (%)	Female (%)	Male (%)	*p*-value	Total (%)	Female (%)	Male (%)	*p*-value	Total (%)	Female (%)	Male (%)	*p*-value	Total (%)	Female (%)	Male (%)	*p*-value
Sunscreen Face	32.5	43.7	22.2	<0.001	34.6	46.6	23.6	<0.001	37.3	50.0	26.1	<0.001	33.2	45.4	21.5	<0.001
Sunscreen Body	26.6	31.6	22.0	<0.001	28.3	32.5	24.6	0.003	30.3	36.9	24.5	<0.001	26.1	31.6	20.8	<0.001
Shirt with sleeves covering shoulders	52.1	49.4	54.6	0.013	53.0	49.2	56.5	0.017	52.2	49.0	55.0	0.049	53.1	49.5	56.5	0.003
Headwear	29.5	20.8	37.5	<0.001	32.7	23.2	41.2	<0.001	31.9	23.2	39.4	<0.001	29.0	20.7	36.9	<0.001
Sunglasses	46.8	49.5	44.4	0.016	47.7	50.3	45.4	0.131	50.3	53.7	47.3	0.030	46.9	49.9	44.1	0.016
Traveling in the shade	38.5	43.8	33.8	<0.001	38.0	41.4	34.9	0.034	39.0	43.9	34.8	<0.001	39.7	44.3	35.4	<0.001

*p*-value based on Chi squared test; we present exploratory unadjusted associations. Shown is the proportion of participants who indicated to use the individual measures often or always. Because participants could report multiple reasons for active travel, the groups are not mutually exclusive and may overlap. Those who travel solely to accompany children, to drop them off or pick them up are excluded from this table.

Table 1 also shows use of sun protection measures by kind of active travel. Wearing a shirt with sleeves covering the shoulders was the most frequently rated sun protection measure in all three subgroups: those traveling to work or an educational facility (53.0%), to leisure activities (52.2%), and to run errands (53.1%). The second most reported behavior was wearing sunglasses (47.7%, 50.3%, and 46.9%), followed by traveling distances in the shade (38.0%, 39.0%, and 39.7%), application of sunscreen on the face (34.6%, 37.3% and 33.2%), wearing headwear (32.7%, 31.9% and 29.0%), and the application of sunscreen on the remaining areas of the body exposed to the sun (28.3%, 30.3% and 26.1%). The subgroup analyses showed the same sex/gender differences as the overall sample; only the difference for wearing sunglasses while traveling to work/educational facility was not statistically significant (*p* = 0.131; Table 1).

### 3.2 Sun protection during active travel with children

Regarding sun protection during active travel with children, we found discrepancies between the personal sun protection and the sun protection of the child/children (Table 2). All comparisons were statistically significant (*p* < 0.001). Use of sun protection measures for children was consistently more prevalent than for participants themselves, except for wearing sunglasses (30.9% vs. 51.1%; *p* < 0.001). The most frequently reported sun protection behavior by caregivers was wearing sunglasses (51.1%), followed by wearing a shirt with sleeves covering the shoulders (48.9%), wearing headwear (38.5%), using sunscreen on the face (37.5%), traveling in the shade (36.1%), and applying sunscreen to remaining areas of the body exposed to the sun (30.7%).

**Table 2:** Sun protection use during active travel with children (*n* = 554).

Sun protection behaviors	Own sun protection				Sun protection of the child/children			
	Total (%)	Female (%)	Male (%)	*p*-value	Total (%)	Female (%)	Male (%)	*p*-value
Sunscreen Face	37.5*	46.7	28.7	<0.001	65.7*	71.3	60.3	0.006
Sunscreen Body	30.7*	33.8	27.7	0.116	65.9*	70.6	61.3	0.022
Shirt with sleeves covering shoulders	48.9*	43.8	53.9	0.017	67.9*	68.8	67.0	0.663
Headwear	38.4*	29.4	47.2	<0.001	69.1*	71.7	66.7	0.201
Sunglasses	51.1*	50.0	52.1	0.616	30.9*	28.7	33.0	0.273
Traveling in the shade	36.1*	37.9	34.4	0.395	50.9*	57.4	44.7	0.003

Shown is the proportion of participants who indicated to use the individual measures often or always.

*pairwise comparison between sun protection of the caregiver and the child/children was significant (*p* < 0.001; McNemar test).

*p*-values based on Chi squared test; we present exploratory unadjusted associations.

In contrast, about two thirds of the participants indicated that children wear headwear (69.1%), shirts with sleeves that cover the shoulders (67.9%), and sunscreen on body (65.9%) and face (65.7%) during active travel. Half of the respondents (50.9%) reported that the children travel in the shade and 30.9% indicated that the children wear sunglasses. Female caregivers were significantly more likely to report sunscreen use on children's faces (71.3% vs. 60.3%, *p* = 0.006) and body (70.6% vs. 61.3%, *p* = 0.022), and to travel in the shade (57.4% vs. 44.7%, *p* = 0.003) compared to male caregivers.

When comparing the use of sun protection in those who were engaged in active travel with children to those who were engaged for other reasons (i.e., to/from work or an educational institution, leisure activities, or errands), only headwear use differed in descriptive comparisons (Figure 1). During active travel with children, 38.5% (95% CI: 34.5%–42.6%) reported wearing headwear, compared with 29.5% (95% CI: 27.9%–31.1%) in their counterparts.

**Figure 1:**
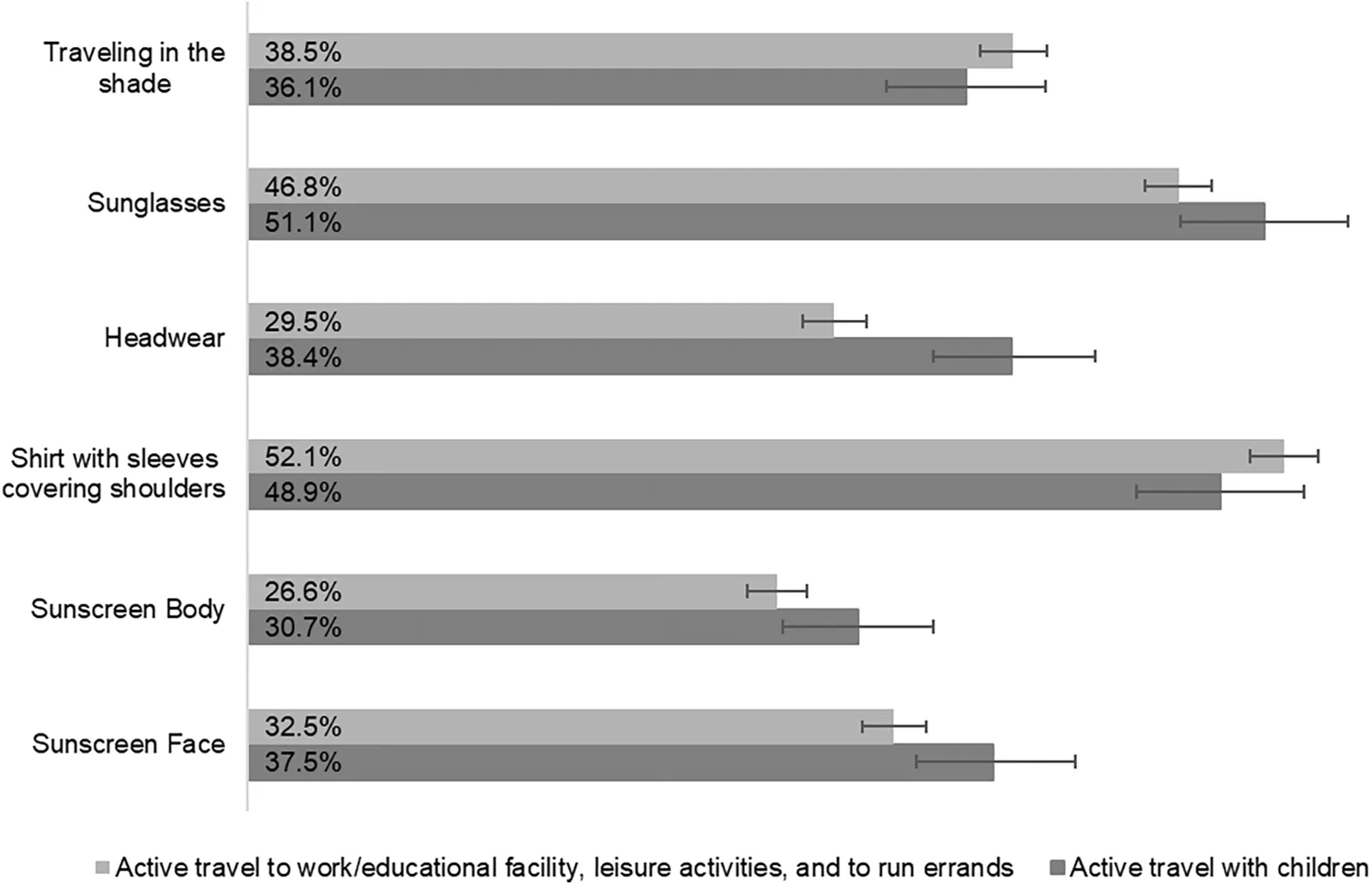
Comparison of sun protection use by kind of travel.

### 3.3 Sun protection by frequency of active travel

We found significant associations between travel frequency and the use of sun protection measures (Table 3). Among participants actively traveling to work, to leisure activities or to run errands, those who traveled frequently were more likely than occasional travelers to apply sunscreen on the face (37.3% vs. 27.1%, *p* < 0.001) and body (28.8% vs. 24.2%, *p* = 0.003). Frequent travelers were also more likely to cover their shoulders with a shirt (56.0% vs. 47.8%, *p* < 0.001), wear headwear (31.1% vs. 27.7%, *p* = 0.040), and wear sunglasses (48.7% vs. 44.8%, *p* = 0.028).

**Table 3:** Sun protection use by frequency of active travel.

Sun protection behaviors	Respondents who travel to or from work, leisure activities or to run errands			Respondents who travel with children			Children of respondents who travel with children		
	Often (*n* = 1,683)	From time to time (*n* = 1,503)	*p*-value	Often (*n* = 320)	From time to time (*n* = 234)	*p*-value	Often (*n* = 320)	From time to time (*n* = 234)	*p*-value
	(%)	(%)		(%)	(%)		(%)	(%)	
Sunscreen Face	37.3	27.1	<0.001	41.6	32.1	0.022	70.3	59.4	0.008
Sunscreen Body	28.8	24.2	0.003	33.1	27.4	0.145	69.1	61.5	0.065
Shirt with sleeves covering shoulders	56.0	47.8	<0.001	52.8	43.6	0.032	73.1	60.7	0.002
Headwear	31.1	27.7	0.040	42.5	32.9	0.022	72.2	65.0	0.069
Sunglasses	48.7	44.8	0.028	52.2	49.6	0.543	33.8	26.9	0.086
Traveling in the shade	39.3	37.7	0.369	39.1	32.1	0.090	55.0	45.3	0.024

*p*-values based on Chi squared test; we present exploratory unadjusted associations. Shown is the proportion of participants who indicated to use the individual measures often or always. Because participants could report multiple reasons for active travel, the groups are not mutually exclusive and may overlap.

Compared with participants who accompanied children only from time to time, those who often accompanied children more frequently reported applying sunscreen to the face (41.6% vs. 32.1%, *p* = 0.022), wearing a shirt with sleeves (52.8% vs. 43.6%, *p* = 0.032), wearing headwear (42.5% vs. 32.9%, *p* = 0.022), and traveling in the shade (39.1% vs. 32.1%, *p* = 0.090). For children they accompanied, frequent accompaniers more often reported sunscreen application to the face (70.3% vs. 59.4%, *p* = 0.008), wearing a shirt with sleeves (73.1% vs. 60.7%, *p* = 0.002), and traveling in the shade (55.0% vs. 45.3%, *p* = 0.024) than occasional accompaniers.

### 3.4 Behavioral adaptation in active travel in response to increased solar radiation

Among all active travelers, 42.8% indicated to decide against walking or cycling due to increased solar radiation in the summer, and instead use the car or bus/train. This behavior was more prevalent in those who travel from time to time compared to those who travel often (50.7% vs. 35.6%, *p* < 0.001). In addition, 53.2% reported using additional sun protection measures due to increased solar radiation. There were no significant differences by travel frequency (54.3% vs. 52.0%, *p* = 0.190).

## 4 Discussion

Sun-protection behaviors were employed during active travel; however, adherence could be improved in both adults and children. Although children were more frequently protected than accompanying adults, their greater vulnerability to UV damage indicated that further improvement is warranted. Participants also reported having modified their active travel behaviors in response to increased solar radiation.

The most frequently employed sun-protection measures were wearing a shirt that covered the shoulders, wearing sunglasses, and traveling in the shade. By contrast, sunscreen application and wearing headwear were less common. Nonetheless, it should be noted that, particularly among men, the use of sleeved shirts likely reflects fashion or practical preferences rather than an explicit sun-protection motive (21). Likewise, headwear during active travel might often have been a bicycle helmet, which can provide some sun protection but is primarily worn for safety. In addition, our findings raise the question, why sunscreen use, especially on the body, is the least common sun protection behavior. Prior research has identified practical barriers (e.g., forgetting, lack of routine, perceived effort) and product-related perceptions (e.g., greasy/sticky texture, unpleasant skin feel) as common reasons for not using sunscreen (34–36). An additional consideration in active travel is that its potential door-to-door time advantage (37) may be substantially reduced or entirely offset by the time required to apply sunscreen. While industry develops more user-friendly sunscreen formulations, education should also emphasize the importance of sun protection for preventing skin cancer, even during short periods of exposure, such as when traveling in everyday life. Beyond individual-level prevention, there may be a need to consider adapting aspects of the physical (built) environment. The infrequent use of shaded routes in our survey may point to a general shortfall in shade provision along pedestrian and cycling corridors, which could be addressed in future urban planning. Accordingly, a dual approach is needed: measures that support sun protection behaviors and structural environmental interventions, particularly, in the context of heat exposure and rising UV exposure as consequences of climate change.

The observed sex/gender differences in sun protection during active travel were also reported for general sun protection: women were more likely to use sunscreen (35, 38–42), whereas men were more likely to rely on clothing (14, 43) and headwear (14, 39, 43). However, these patterns may also reflect factors other than sun protection *per se*: in summer, women more often wear sleeveless tops than men, and men more frequently wear headwear (e.g., caps) compared to women. However, sex/gender of the participant is not only significantly related to their own use of sun protection measures, but also to their children's sun protection. Women were more likely to report sun protection measures for the accompanied children than men. This association of children's sun protection with caregiver's sex/gender was also observed in earlier studies (44, 45). However, children's age (20, 46) and sex/gender (44, 47, 48) also play a role, which we were not able to examine in the context of active travel in this study.

The least common sun protection behavior for children was wearing sunglasses. This has also been observed in other German population-based studies that examined general sun protection among children (16). Reasons could include a lack of knowledge about the potential UV-related damage to children's eyes (49) or that children's sunglasses are impractical for everyday usage. As the reasons for (not) using the individual sun protection measures were not within the focus of our survey, further studies should address this aspect to improve sunglass use.

Frequent active travelers were more likely than occasional travelers to use sunscreen and clothing for sun protection. This is encouraging, as those more frequently exposed during active travel were also more likely to adopt protective measures. Nevertheless, overall use of sun protection could be improved, and individuals who engage in active travel only occasionally also need to protect themselves using sun protection measures.

Approximately four in ten active travelers chose not to walk or cycle when they perceived that solar radiation was elevated in summer, which is favorable from a UV exposure standpoint. However, differences emerged between frequent and occasional active travelers; in particular, those who engaged in active travel more regularly would benefit from targeted education and awareness efforts to further promote sun-protection practices under high-UV conditions. It should be emphasized that, from a prevention standpoint, active travel is highly desirable for reducing the burden of chronic, non-communicable diseases (50, 51). We observe a trade-off between two health-relevant behaviors: sun protection and active travel (52). A complete avoidance of active travel in summer is not a viable alternative and instead underscores the critical role of natural and built shade provision along active travel routes.

### 4.1 Limitations

Our study used nationwide data encompassing a large case number and addressed an innovative research question, with no comparable investigations to date. However, several potential limitations of this study should be taken into account. The underlying data was collected via self-report in November. Therefore, social desirability and recall bias cannot be ruled out. We used a quota sampling based on sex/gender, age, education, and federal state, nonetheless, this limits nationwide generalizability due to potential selection bias. Given the pilot nature of the study, we could not always use validated scales and therefore developed new items, which we evaluated in a comprehensive cognitive pretest with 15 participants.

In addition, more detailed analyses of sun-protection behaviors during active travel with children are needed. This study is only a starting point; child-specific characteristics [e.g., age (20, 46)] likely play a role but could not be accounted for here given the pilot nature of the study. We asked only in general whether participants traveled with a child or children and to what extent the child(ren) used sun-protection measures. In addition, results may be influenced by confounding, warranting more thorough investigation in future work.

Moreover, we deliberately excluded leisure activity from our analyses, because our focus was on walking/cycling for transport and not on walking/cycling for recreation (30). However, sun-protection behaviors during recreational walking or cycling (e.g., dog walking) should be examined in future studies. Future studies should also examine travel duration in relation to UV protection. In this initial study, we defined active travel as >10 min and could not further quantify UV exposure, which may have introduced exposure heterogeneity. It will be important to assess whether sun-protection behavior varies by travel duration.

### 4.2 Conclusion

Our data indicate that, although sun-protection measures are used during active travel, there may be room for improvement. In particular, sunscreen use on body sites not covered by clothing warrants strengthening. While skin cancer prevention campaigns often target prolonged sun exposure (e.g., during beach or mountain vacations), there is considerable untapped potential for education around everyday trips. Even comparatively short exposures, such as a 20-minute bicycle ride to work at a time of day when UV levels are high, can cause sunburn and increase the skin cancer risk. These routine exposures should therefore be explicitly addressed in future skin cancer prevention messaging and guidance on appropriate sun protection.

## Data Availability

The raw data supporting the conclusions of this article will be made available by the authors, without undue reservation.
