# Supplementary material for: Sun protection during active travel on foot or by (e-)bike: an exploratory nationwide cross-sectional study from Germany
Source: Front Sports Act Living. 2026 Oct 14;8:1957202. doi: 10.3389/fspor.2026.1957202 (PMC13713808; doi:10.3389/fspor.2026.1957202)
Supplement: Supplementary file 1 [file Datasheet1.docx]

Supplementary Material

Figure S1: Overview of participants by travel behavior

Legend: For the question on reasons for everyday traveling, multiple answers were allowed. Respondents whose sole reason(s) were leisure activity or walking the dog were excluded from subsequent analyses.

Table S1: Characteristics of participants engaged in active travel for transport reasons

|  | All travelers | | Those who travel to or from work, leisure activities, or to run errands | | Those who travel with children | |
| --- | --- | --- | --- | --- | --- | --- |
|  | (n=3,264) | | (n=3,186) | | (n=554) | |
|  | n | % | n | % | n | % |
| **Sex** | 3,264 |  | 3,186 |  | 554 |  |
| Female | 1,557 | 47.7% | 1,517 | 47.6% | 272 | 49.1% |
| Male | 1,696 | 52.0% | 1,658 | 52.0% | 282 | 50.9% |
| Diverse | 11 | 0.3% | 11 | 0.4% | 0 | 0.0% |
| **Age** | 3,264 |  | 3,186 |  | 554 |  |
| Mean (SD) | 42.36 (13.6) | | 42.46 (13.7) | | 40.28 (10.4) | |
| **Immigrant background** | 3,264 |  | 3,186 |  | 554 |  |
| No | 2,968 | 90.9% | 2,901 | 91.1% | 497 | 89.7% |
| Yes | 296 | 9.1% | 285 | 9.0% | 57 | 10.3% |
| **Education** | 3,219 |  | 3,142 |  | 544 |  |
| Low | 781 | 24.3% | 766 | 24.4% | 97 | 17.8% |
| Medium | 989 | 30.7% | 957 | 30.5% | 191 | 35.1% |
| High | 1,449 | 45.0% | 1,419 | 45.2% | 256 | 47.1% |
| **Occupation** | 3,236 |  | 3,158 |  | 552 |  |
| Fulltime | 1,886 | 58.3% | 1,842 | 58.3% | 328 | 59.4% |
| Parttime | 620 | 19.2% | 599 | 19.0% | 139 | 25.2% |
| None | 730 | 22.6% | 717 | 22.7% | 85 | 15.4% |
| **Partnership** | 3,251 |  | 3,173 |  | 553 |  |
| No | 1,109 | 34.1% | 1,103 | 34.8% | 82 | 14.8% |
| Yes | 2,142 | 65.9% | 2,070 | 65.2% | 471 | 85.2% |
| **Skin type** | 3,264 |  | 3,186 |  | 554 |  |
| I-II | 1,324 | 40.6% | 1,289 | 40.5% | 223 | 40.3% |
| III-IV | 1,940 | 59.4% | 1,897 | 59.5% | 331 | 59.8% |

Due to rounding numbers may not always add up to 100%; SD=Standard Deviation

Because participants could report multiple reasons for commuting, the groups are not mutually exclusive and may overlap
